# Silencing the ASI gustatory neuron pair extends lifespan

**DOI:** 10.17912/ft9e-7e37

**Published:** 2018-06-01

**Authors:** Peter Chisnell, Cynthia Kenyon

**Affiliations:** 1 Department of Biochemistry & Biophysics, University of California, San Francisco, San Francisco, CA 94143-2200, USA

**Figure 1.**
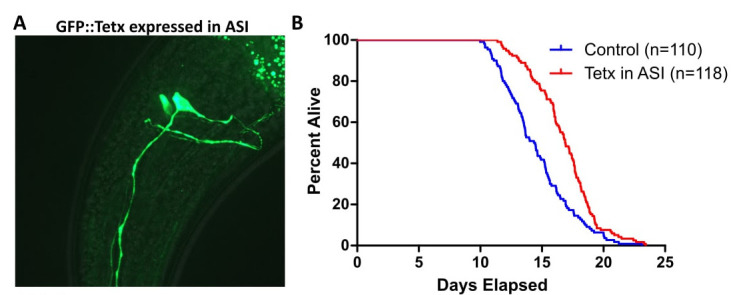


## Description

Disrupting the function of sensory neurons of *C. elegans* can increase their lifespan (Apeld and Kenyon 1999). This effect is not limited to large-scale disruption, as ablation of single pairs of neurons have been shown to modify lifespan (Alcedo and Kenyon 2004; Lee and Kenyon 2009; Liu and Cai 2013). We tested whether silencing the neuron pair ASI with the tetanus toxin light chain (Tetx), as opposed to ablating it, could increase lifespan. Tetanus toxin disrupts neurotransmission by blocking the release of both small clear-core vesicles and large dense-core vesicles, but should not affect communication via gap junctions (Schiavo et al. 1992; McMahon et al. 1992). We expressed GFP::Tetx using the ASI-specific promoter p*gpa-4* (Figure Panel A) and conducted lifespan assays comparing animals with high fluorescence and undetectable fluorescence. Tetx in ASI extended lifespan in otherwise wild-type animals (Figure Panel B, Table 1, 14.9% average median lifespan increase across 5 replicates).

**Table 1**

**Table:** 

**Experiment #**	**Strain**	**Median Lifespan**	**Sample Size**	**P (Tetx vs. Control)**	**Automated?**
1	Tetx in ASI	17	66	0.0125	no
	Control	15	55		
2	Tetx in ASI	16.9	118	<0.0001	yes
	Control	14.4	110		
3	Tetx in ASI	15.9	141	<0.0001	yes
	Control	13.4	93		
4	Tetx in ASI	16.3	156	0.0471	yes
	Control	14.1	86		
5	Tetx in ASI	23	98	0.0025	no
	Control	21	68		

## Methods

Lifespan assays were conducted as previously described (Apfeld and Kenyon 1999) by hand with no FUDR, as well as utilizing automated lifespan machines (Stroustrup et al. 2013).

## Reagents

Strains: CF4126: *muEx641*[pPC30(p*gpa-4*::GFP::Tetx) + p*unc-122*::RFP]
